# Identification of Ferroptosis‐Related Hub Genes as Diagnosis Biomarkers and Therapeutic Monitoring for Major Depressive Disorder Diagnosis

**DOI:** 10.1049/syb2.70045

**Published:** 2025-11-27

**Authors:** Shenghui Huang, Shoupin Xie, Fei Feng, Yanyan Wan, Yanping Ma, Yafeng Wang, Fan Zhang, Xinhong Chen, Ping Tang, Hailong Li

**Affiliations:** ^1^ Department of Mental Health and Sleep Center Gansu Provincial Hospital of Traditional Chinese Medicine Lanzhou Gansu China; ^2^ Department of Neurology Lanzhou First People's Hospital Lanzhou Gansu China; ^3^ Department of Clinical Psychology (Psychosomatic Medicine) Shenzhen People's Hospital Shenzhen China; ^4^ Department of Science and Education Luohu District People's Hospital Shenzhen China; ^5^ Department of Geriatrics Affiliated Hospital of Gansu University of Traditional Chinese Medicine Lanzhou China; ^6^ Department of General Practice, Luohu Clinical College, School of Medicine Shantou University Shenzhen China; ^7^ Department of Geriatrics Shenzhen Integrated Traditional Chinese and Western Medicine Hospital Shenzhen Guangdong China; ^8^ Shenzhen Clinical Medical College of Integrated Traditional Chinese and Western Medicine Guangzhou University of Chinese Medicine Shenzhen China

**Keywords:** biocomputers, bioinformatics, data analysis, data mining

## Abstract

Major Depressive Disorder (MDD) is linked to increased neurodegenerative risk. Emerging evidence implicates ferroptosis in neuropsychiatric disorders, prompting investigation of its role in MDD through key gene identification. Three microarray datasets from the GEO database were analysed. Weighted gene co‐expression network analysis (WGCNA) identified MDD‐related module genes (MRGs) while ferroptosis‐related genes (FRGs) were extracted from the FerrDb database. Overlapping genes between MRGs and FRGs were prioritised for mechanistic exploration. Functional enrichment (GO/KEGG) and protein‐protein interaction (PPI) network analyses (via Cytoscape and CytoHubba) highlighted hub genes. Machine learning algorithms were applied to develop a diagnostic model, validated through nomogram analysis, calibration curves, decision curve analysis (DCA), ROC curves (AUC evaluation), gene set enrichment analysis (GSEA), and DGIdb‐based drug prediction. Differential expression analysis identified 1878 MDD‐associated genes (715 downregulated, 1163 upregulated). Four FRGs—MAPK14, WIPI1, DUSP1, and ULK1—emerged as diagnostic biomarkers, showing significant immune cell infiltration correlations (e.g., neutrophils, dendritic cells) and enrichment in pathways like MAPK signalling. The study highlights ferroptosis‐related genes (ULK1, MAPK14, WIPI1, DUSP1) as potential diagnostic and therapeutic targets in MDD, linked to neuroimmune interactions and cellular stress responses. These findings underscore MDD's pathophysiological complexity and may guide strategies for managing MDD and neurodegenerative comorbidities.

## Graphical abstract

This study explored ferroptosis involvement in major depressive disorder (MDD) by integrating GEO microarray data, WGCNA, and ferroptosis‐related gene analysis, identifying four key genes (MAPK14, WIPI1, DUSP1, ULK1) as diagnostic biomarkers. Functional and interactome analyses linked these genes to neuroimmune interactions and cellular stress pathways, suggesting their potential as MDD therapeutic targets.

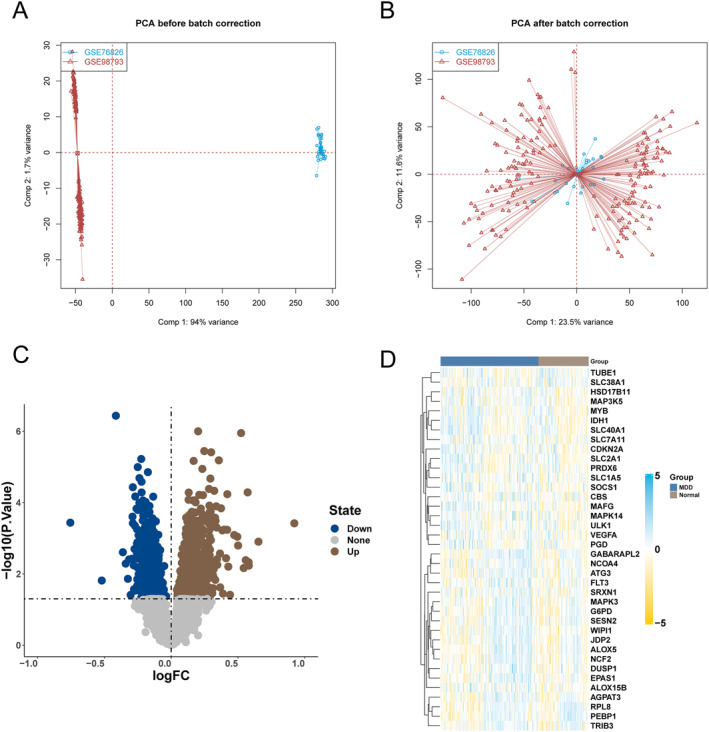

## 1 Introduction

Major depressive disorder (MDD) is a serious mental health disorder that has significant impacts on individuals and society worldwide. In China, according to the latest epidemiological data, the lifetime prevalence of depressive disorders is 6.8%, with depression accounting for 3.4%. In the USA, the lifetime prevalence of MDD is approximately 17% for men, 30% for women, and 9% among the general adult population [1]. MDD is more prevalent in women than men, unemployed people than employed, and those who were separated, widowed, or divorced than people who were married or cohabiting [2]. Despite the rising global prevalence of depression, treatment rates remain alarmingly low, with only 9.5% of patients receiving any form of treatment and a mere 0.5% achieving full therapeutic adherence. The global pandemic of COVID‐19 has also aggravated mental health problems, leading to a significant increase in the incidence rate of depression and anxiety. The prevalence of MDD among COVID‐19 survivors was reported as 31% [3]. In addition, the prevalence of depression among adolescents and students is also on the rise. The situation of prevention and control faced a new challenge.

Major Depressive Disorder (MDD) is a debilitating psychiatric disorder with complex pathophysiology, where emerging evidence suggests ferroptosis—an iron‐dependent form of regulated cell death—may contribute to its neurobiological mechanisms [4, 5]. Recent studies have implicated ferroptosis in neuronal damage and immune dysregulation, offering potential insights into MDD diagnosis and treatment [6]. Recent studies have used gene expression profiling to discover ferroptosis‐related genes (FRGs) that could be diagnostic indicators for MDD, such as, in vitro investigations demonstrated that ROS buildup and decreased expression of SLC7A11 and GPX4 were identified in the CORT‐induced depression‐like cell model, indicating that ferroptosis was implicated in neuronal damage [7]. Researchers identified a group of FRGs with differential expression in MDD patients compared with healthy controls using information from the Gene Expression Omnibus (GEO) database [4]. Notably, a signature comprising three FRGs, ALOX15 B, RPLP0, and HP—has exhibited significant diagnostic potential, with a receiver operating characteristic (ROC) ROC analysis validated its discriminative power to identify MDD patients with healthy individuals with an AUC of 0.783. Beyond their role as biomarkers, these FRGs are intricately linked to immune dysregulation, which is a critical component in the pathophysiology of MDD. Gene ontology (GO) enrichment analysis has provided insight on the functions of these genes in immune‐related pathways. Furthermore, the association between these FRGs and immune cell infiltration in MDD shows a complicated interplay between ferroptosis and immune system modulation, which could influence the disease's course. Understanding ferroptosis in MDD brings up new options of therapeutic intervention. By focussing on dysregulated ferroptotic circuits, new therapeutics that complement or even outperform conventional antidepressant therapies may be developed. The involvement of FRGs as not only diagnostic markers but also therapeutic targets highlight the importance of continued research in this field.

In this paper, we decided to incorporate ferroptosis‐related indicators into the diagnostic and treatment strategy for MDD to develop more specific focused therapies that can greatly improve patient outcomes.

## 2 Materials and Methods

### 2.1 Expression Spectrum Data Downloading and Processing

The severe Major depression dataset (MDD) with data numbers GSE98793, GSE76826, GSE32280, GSE39653, and GSE52790 was downloaded from the database NCBI (Gene Expression Omnibus, GEO; http://www.ncbi.nlm.nih.gov/geo/). GSE98793 retained 192 samples (MDD = 128, Normal = 64); GSE76826 maintained 32 samples (MDD = 20, Normal = 12); GSE32280 retained 16 samples (MDD = 8, Normal = 8); GSE39653 retained 45 samples (MDD = 21, Normal = 24); and GSE52790 retained 22 samples (MDD = 10, Normal = 12). Following the removal of batch effects with the SVA package (https://www.bioconductor.org/packages/release/bioc/html/sva.html), the GSE98793 and GSE76826 datasets were utilised as training sets, whereas the GSE32280, GSE39653, and GSE52790 datasets were used as validation. The probe expression matrix was obtained, processed, and standardised, and the associated platform annotation file was downloaded concurrently to transform the probes into gene symbols. For different probes corresponding to the same gene symbol, the average value is taken as the expression value of the gene for subsequent analysis.

### 2.2 Identification of Differentially Expressed Genes and WGCNA Analysis

First, the limma package (Version: 3.50.0, https://bioconductor.org/packages/release/bioc/html/limma.html) [8] was used to perform MDD VS normal differential expression analysis on the training set. When ensuring sufficient differentially expressed genes are obtained, the threshold was improved as much as possible. Therefore, the final threshold is set to log_2_ |FC| > 0 with *p* < 0.05. The WGCNA package (Version: 1.71, https://cran.r‐project.org/web/packages/WGCNA/index.html) [9] was used to perform WGCNA analysis on disease samples in the training set. A total of 212 samples remained after 12 samples with a pruning tree height of 30 were eliminated from the training set. A soft threshold of 1–20 was used for topological computations. The relationship matrix was converted into an adjacent matrix, which was further converted into a topological overlap matrix (TOM) for average link hierarchical clustering, based on the ideal soft threshold (*β* = 5). Based on TOM, relevant modules were categorised, and each module had at least 30 genes. After that, related modules were combined (pruning height = 0.25). The merged module's association with the molecular subtypes of necroptosis genes can be determined using Pearson's approach; the core module is the one with the highest correlation.

### 2.3 Identification of Differentially Expressed Ferroptosis‐Related Genes

Ferroptosis‐related genes were retrieved from the FerrDb database (http://www.zhounan.org/ferrdb) [10]. Afterwards, Pearson correlation analysis was performed on the intersection genes (differential ferroptosis‐related genes) between module genes and ferroptosis‐related genes in the GEO training set. The clusterProfiler package [11] (Version: 4.2.2, https://bioconductor.org/packages/release/bioc/html/clusterProfiler.html) was utilised. GO and KEGG enrichment analyses were carried out concurrently on differentially expressed ferroptosis genes in order to determine the biological pathways connected to these genes. Then, a protein‐protein interaction network for differentially expressed ferroptosis‐related genes was constructed using these data and added to the STRING database (https://cn.string‐db.org) [12]. To predict and examine the top 10 important genes in the PPI network, the cytoHubba plugin in Cytoscape along with the topology was used to analyse techniques MCC, MNC, Degree, and EPC. Genes at the 10 gene junctions.

### 2.4 Construction of Hub Gene of the MDD Diagnostic Model

The training set data were subjected to the LASSO algorithm using the glmnet package (Version 4.1‐4, https://cran.r‐project.org/web/packages/glmnet/index.html) [13], the SVM‐RFE algorithm using the e1071 package (Version: 1.7‐13, https://cran.r‐project.org/web/packages/e1071/index.html), and the RF algorithm using the randomForest package (Version 4.7‐1.1, https://cran.r‐project.org/web/packages/randomForest/index.html). These three algorithms were used to screen feature genes from hub genes, and the intersection genes (diagnostic genes) chosen from the outcomes of the three algorithms were used in the RMS package (Version: 6.5‐0, https://cran.r‐project.org/web/packages/rms/index.html) to construct an MDD diagnostic model using multiple factor logistic regression. The diagnostic score was computed using the following formula:

Riskscore=∑βgene×Expgene

The gene in this instance is represented by the symbol β gene, and Expgene displays the gene's expression level in the training set. To verify the accuracy of the model, the diagnostic score values for each sample in the validation set were also calculated using the same regression coefficients in compliance with the diagnostic score calculation algorithm. The diagnostic model's effectiveness was evaluated using box plots and ROC curves.

### 2.5 Construction of a Diagnostic Nomogram

To facilitate the practical application of diagnostic models, a gene diagnosis nomogram was constructed based on the expression data of diagnostic genes. By drawing a nomogram, the results of the diagnostic were made clearer. Simultaneously, the correction curve and decision curve analysis (DCA) were drawn to demonstrate the accuracy of the model.

### 2.6 Microenvironment Evaluation and Molecular Mechanism Study of MDD and Normal Samples

To further explore the differences in microenvironment (TME) between the MDD and normal samples in the training set, the CIBERSORT algorithm [14] (https://cibersortx.stanford.edu/) was used to determine the scores of 22 immune cells. The Wilcoxon rank‐sum test was used to evaluate differences in immune cells. And the Spearman correlation coefficient is used to evaluate the correlation between diagnostic genes and immune cells. In addition, the GSEA analysis between MDD samples and normal samples was also conducted to identify pathways with significant differential enrichment between subtypes.

### 2.7 PPI Construction of Diagnostic Genes and Prediction of Small‐Molecule Drugs

The protein‐protein interaction analysis was conducted on genes involved in gene interaction to predict colocalisation using the GeneMANIA database, shared protein domains, coexpression, prediction, and correlation between pathways. Additionally, the DGIdb database (https://dgidb.org/) was utilised to predict and diagnose drug interactions between genes.

## 3 Result

### 3.1 Analysis of Expression Patterns of Differentially Expressed Ferroptosis‐Related Genes

**FIGURE 1:**
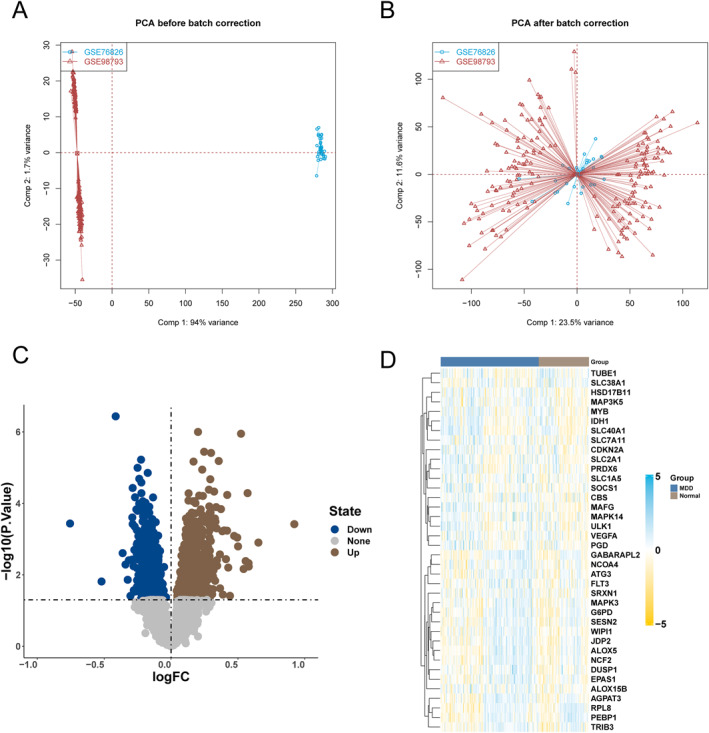
Analysis of expression patterns of differentially expressed ferroptosis genes. (A) PCA scatter plots of GSE98793 and GSE76826 datasets before removing batch effects. (B) PCA scatter plots of GSE98793 and GSE76826 datasets after removing batch effects. (C) Volcano plot analysis of differential expression between MDD and Normal samples in the training set, with brown indicating upregulation and blue indicating downregulation. (D) Differential expression heatmap of ferroptosis genes between MDD and Normal samples.

**FIGURE 2:**
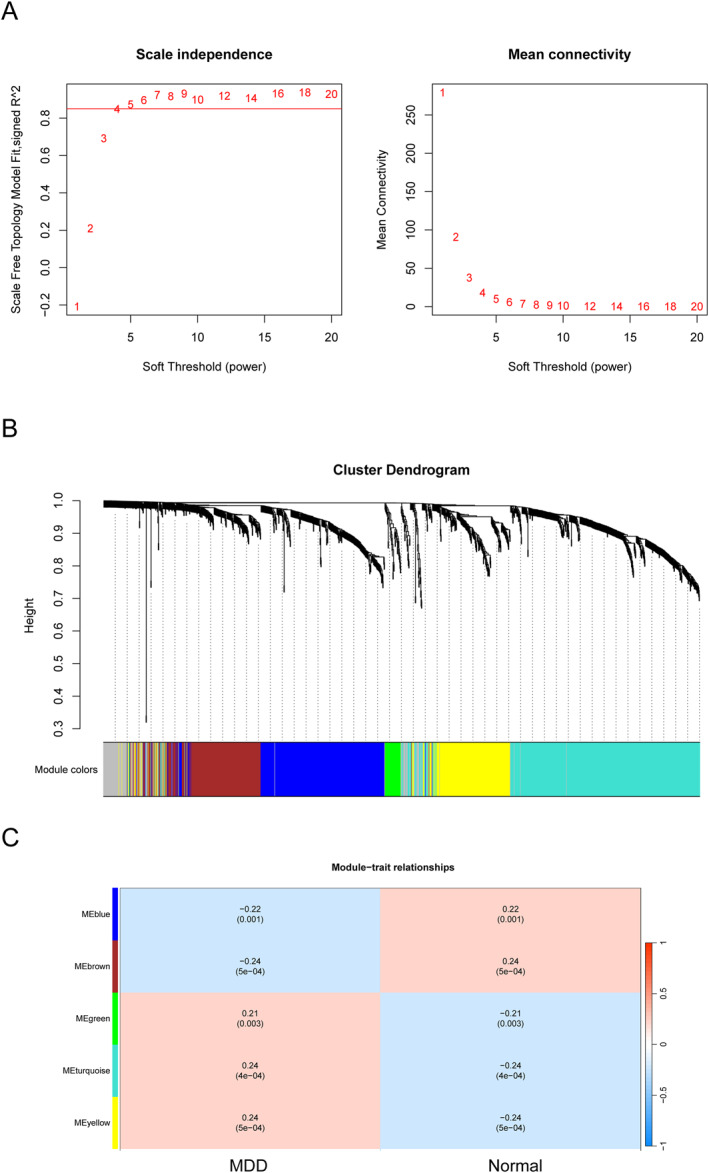
Identification of differentially expressed genes and WGCNA analysis. (A) Scatter plot of coefficients and average connectivity between soft thresholding and scale‐free networks. (B) Waterfall plot of module colour and clustering tree height. (C) The correlation heatmap between modules and disease phenotypes, with red representing positive correlation and blue representing negative correlation.

Firstly, a de‐batch effect was performed on the training set data, and after removing batches, it was observed that there was almost no difference in the samples between the datasets, as was shown in Figure 1A,B. Then, we performed gene differential expression analysis between the MDD and normal samples in the training set, as was shown in Figure 1C, and obtained 1878 differentially expressed genes, of which 715 were down‐regulated and 1163 were up‐regulated. WGCNA analysis was performed based on the expression profile data of these differentially expressed genes. Every sample has strong clustering performance and no outliers. Based on topological calculations, a soft threshold ranging from 1 to 20 was used; and the optimal soft threshold was determined to be 5, as was shown in Figure 2A. According to the soft threshold, the relationship matrix is transformed into an adjacent matrix, which is then transformed into a topological overlap matrix (TOM) for average link hierarchical clustering. The relevant modules are classified based on TOM, and the number of genes in each module is not less than 30, as was shown in Figure 2B. Finally, similar gene modules are merged, and six modules are identified, as shown in Figure 2C. In addition, by calculating the correlation between genes and clinical traits within the module, the module with the highest correlation was selected with a *p*‐value less than 0.05. A turquoise module containing 470 genes was discovered, along with the brown module of 218 genes and the yellow module of 208, which have the highest correlation with disease occurrence.

**FIGURE 3:**
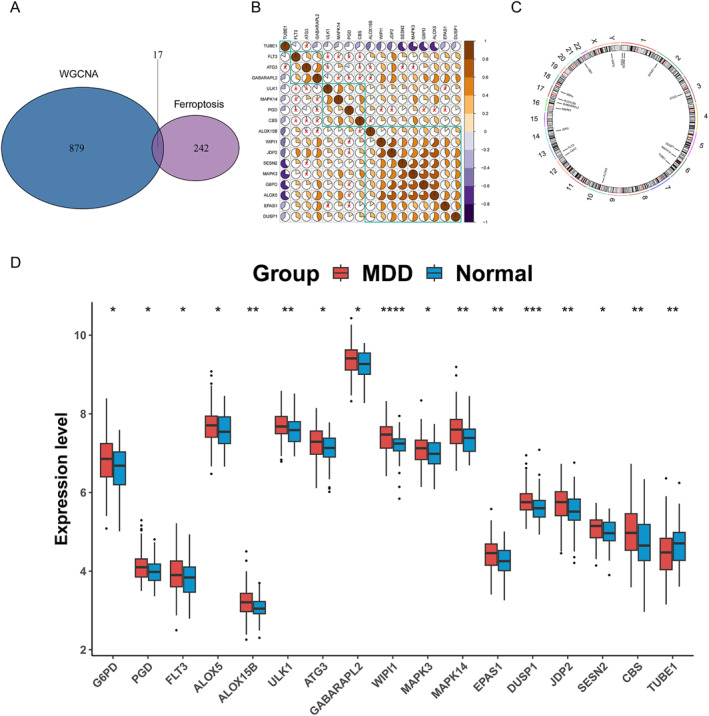
Acquisition of hub genes and identification of diagnostic genes. (A) Intersection Venn diagram of module genes and ferroptosis genes. (B) The correlation heatmap of 17 differentially expressed ferroptosis‐related genes, with brown indicating positive correlation and purple indicating negative correlation. (C) Distribution map of 17 differentially expressed ferroptosis genes on chromosomes. (D) Box plot of 17 differentially expressed ferroptosis genes between MDD and Normal samples in the training set.

These three modules are used as the core modules. Next, these module genes will be intersected with ferroptosis‐related genes to obtain 17 differentially expressed ferroptosis genes, such as G6PD, PGD, FLT3, ALOX5, ALOX15 B, ULK1, ATG3, GABARAPL2, WIPI1, MAPK3, MAPK14, EPAS1, DUSP1, JDP2, SESN2, CBS, and TUBE1, as shown in Figure 3A. The heatmap of the expression values of 17 differentially expressed ferroptosis‐related genes between MDD and normal samples showed different expression patterns, and the correlation analysis results was shown in Figure 3B. Most genes were significantly correlated, indicating that these genes may jointly regulate certain biological processes of MDD. These differentially expressed ferroptosis genes exhibit a wide distribution on chromosomes, as was shown in Figure 3C, and the differential box plot further confirms that the 16 out of 17 differentially expressed ferroptosis genes exhibit significant upregulated in MDD than normal samples, only TUBE1 downregulated in MDD than normal samples.

### 3.2 GO and KEGG Enrichment Analysis of Differentially Expressed Ferroptosis‐Related Genes

**FIGURE 4:**
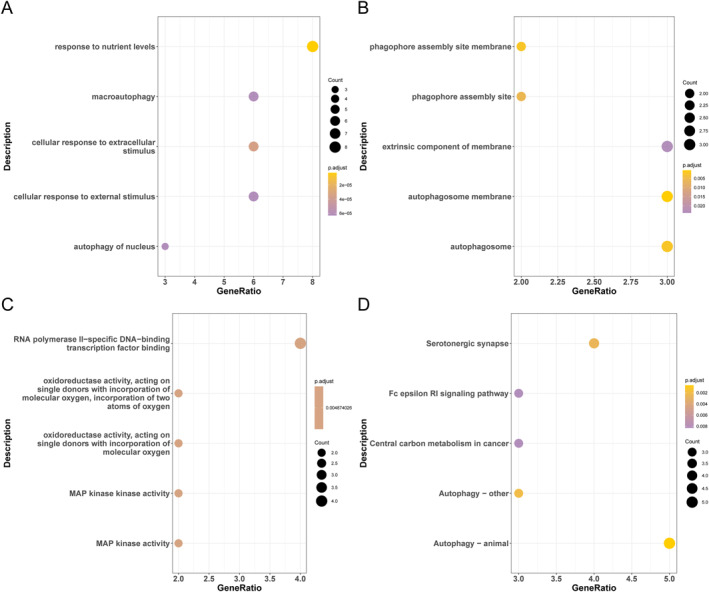
GO and KEGG enrichment analysis of differentially expressed ferroptosis‐related genes. (A) Enrichment analysis bubble plot of biological processes (BP) in GO. (B) Bubble plot of enrichment analysis of cellular components (CC) in GO. (C) Enrichment analysis bubble plot of molecular components (MF) in GO. (D) KEGG's enrichment analysis bubble plot. The size of the bubble represents the gene book enriched in this pathway, with a yellow colour indicating a smaller corrected *p*‐value, a purple colour indicating a larger corrected *p*‐value.

These differential ferroptosis‐related genes were subjected to enrichment studies in order to look into the molecular mechanisms underlying them. These genes were primarily enriched in pathways related to macroautophagy and the response to nutritional levels in BP, according to the GO enrichment data, as was shown in Figure 4A. The attachment, phagophore assembly site, and autophagosome routes in CC, as was shown in Figure 4B, demonstrate the BP enrichment results. The enrichment results of CC for MAP kinase activity in MF, differential ferroptosis‐related genes, and other pathways, as was shown in Figure 4C. These genes were primarily abundant in pathways such as the Fc epsilon RI signalling pathway and the serotonergic synapse, according to the KEGG enrichment data, as was shown in Figure 4D.

### 3.3 Acquisition of Hub Genes and Identification of Diagnostic Genes

**FIGURE 5:**
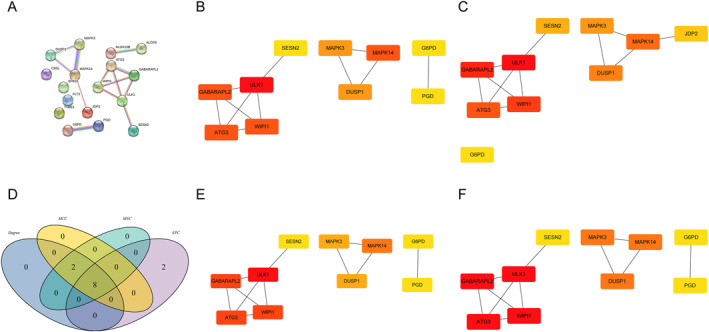
Construction of hub gene of the MDD diagnostic model. (A) Protein‐protein interaction network of 17 differentially expressed ferroptosis‐related genes. (B) The top 10 hub genes selected by the Degree algorithm. (C) The top 10 hub genes selected by the EPC algorithm. (D) The intersection Venn diagram of hub genes obtained by four algorithms. (E) The top 10 hub genes selected by MCC algorithm. (F) The top 10 hub genes selected by MNC algorithm.

**FIGURE 6:**
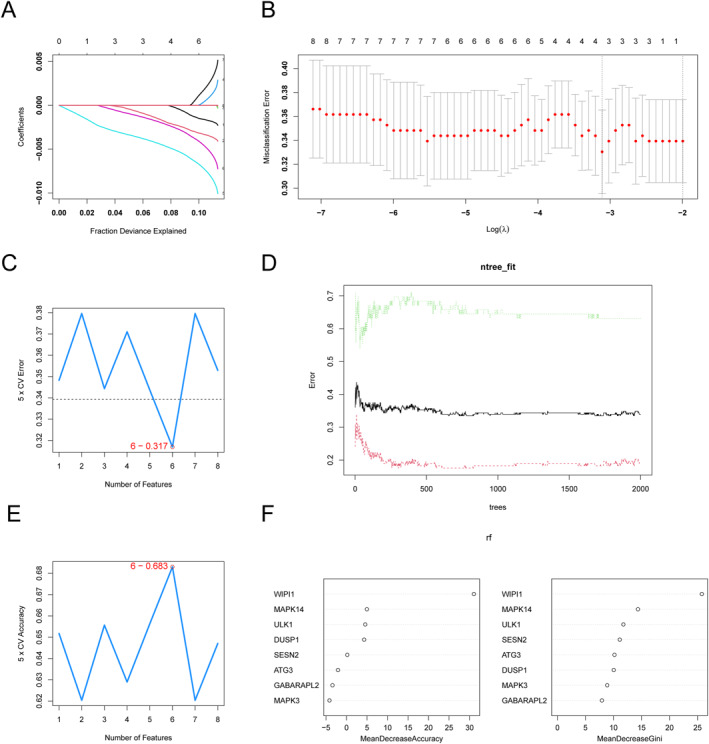
Machine learning screening of diagnostic genes 6 diagnostic genes. (A) LASSO regression analysis variable coefficient value variation curve. (B) Scatter plot of the relationship between LASSO regression analysis cross validation error and log (*λ*) value. (C) Line graph showing the relationship between 5‐fold cross validation error rate and the number of feature genes in the SVM‐RFE algorithm. (D) Line graph showing the relationship between the number of decision trees and error rate in the Rf algorithm. (E) Line graph showing the relationship between 5‐fold cross validation accuracy and the number of feature genes in the SVM‐RFE algorithm. (F) Scatter plot of gene importance in RF algorithm.

First, a protein‐protein interaction network of 17 differentially expressed ferroptosis‐related genes was constructed, as was shown in Figure 5A, and strong interactions were found within these genes. Then, based on the Degree (Figure 5B), EPC (Figure 5C), MCC (Figure 5E), and MNC (Figure 5F) algorithms, the top 10 hub genes were obtained, and the intersection of hub genes from these four algorithms resulted in 8 hub genes, including ULK1, MAPK14, ATG3, GABARAPL2, WIPI1, DUSP1, MAPK3, and SESN2 (Figure 5D). Then, based on these eight hub genes, the diagnostic genes were screened in the training set. The four diagnostic genes, namely ULK1, MAPK14, WIPI1, and DUSP1, were obtained using the LASSO algorithm (Figure 6A,B). Machine learning screening of diagnostic genes 6 diagnostic genes (Figure 6C,E), namely WIPI1, MAPK14, ATG3, DUSP1, MAPK3, and ULK1, were obtained through the SVM‐RFE algorithm. Finally, 6 diagnostic genes (Figure 6D,F), namely ULK1, MAPK14, ATG3, WIPI1, DUSP1, and SESN2, were obtained through the RF algorithm.

### 3.4 Construction of a Diagnostic Model

**FIGURE 7:**
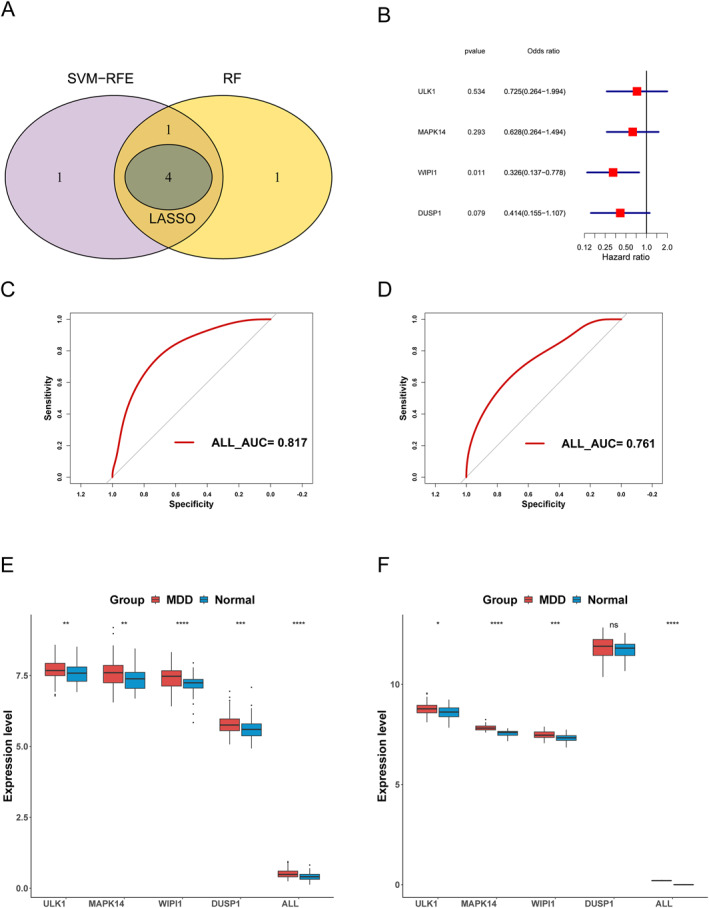
Construction of a diagnostic model. (A) Venn diagram of the intersection of diagnostic genes obtained by LASSO, SVM‐RFE, and RF algorithms. (B) Multi factor Rogers regression forest plot. (C) ROC curve for diagnosing genes and diagnostic scores in the training set. (D) Validation of the ROC curve of centralised diagnostic genes and diagnostic scores. (E) Box plot showing the differences in gene expression levels and diagnostic scores between MDD and Normal samples in the training set. (F) Verification of the box plot of the difference between diagnostic genes and diagnostic scores in MDD and Normal samples.

**FIGURE 8:**
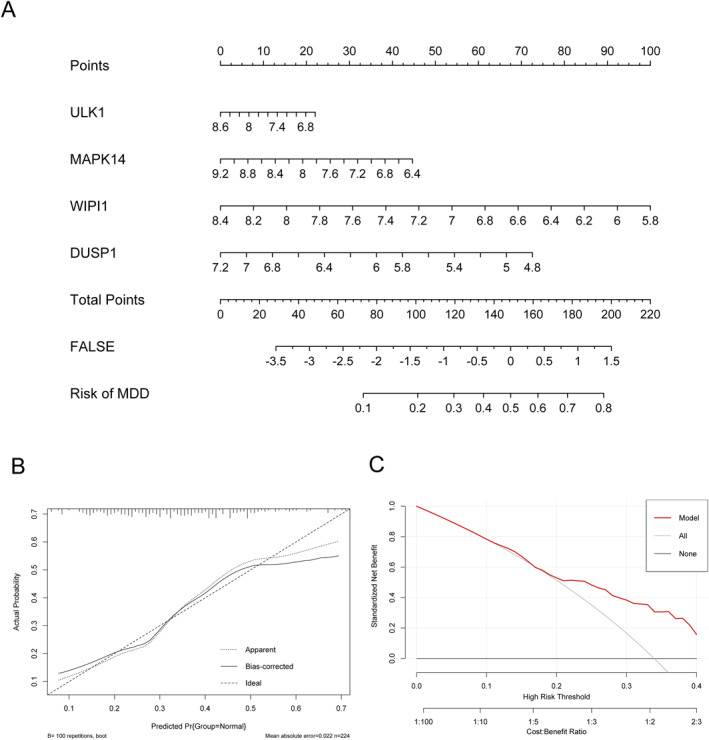
Construction of a diagnostic nomogram. (A) Nomogram diagram. (B) Nomogram calibration curve. (C) DCA of Nomogram.

The intersection of diagnostic genes was obtained from the above three algorithms, totalling four diagnostic genes, namely ULK1, MAPK14, WIPI1, and DUSP1, as were shown in Figure 7A, and constructing an MDD diagnostic model through multi‐factor logistic regression. In the training set, the AUC value of the constructed model's ROC curve was found to be greater than 0.7, as was shown in Figure 7C, and its performance was significantly better than the predictive performance of a single diagnostic gene, indicating favourable predictive performance. At the same time, there was a significant difference in diagnostic scores and most diagnostic genes between MDD and normal samples, as was shown in Figure 7E. To verify the results of the training set, ROC curve analysis on the validation set was also conducted. The results showed that the diagnostic model AUC value in the validation set was still greater than 0.7, as shown in Figure 7D, which was significantly better than the predictive performance of a single diagnostic gene. Moreover, the diagnostic score and gene expression value still showed significant differences between the MDD sample and the normal sample, as was shown in Figure 7F. Next, a nomogram as shown in Figure 8A, a calibration curve as shown in Figure 8B, and a DCA curve as shown in Figure 8C were drawn based on diagnostic genes, all of which demonstrate favourable predictive accuracy of the nomogram.

### 3.5 Microenvironment Assessment and Molecular Mechanism Study

**FIGURE 9:**
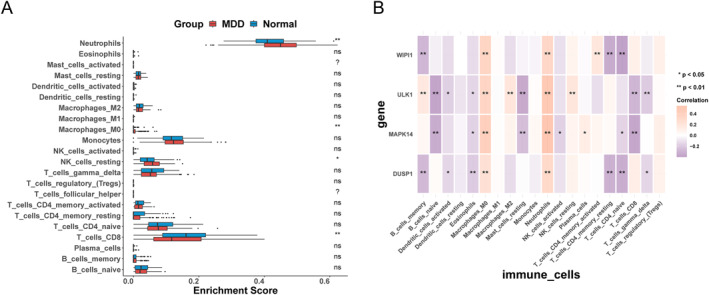
Microenvironment assessment. (A) Box plot of differences in immune cell infiltration levels between MDD and Normal samples evaluated by the CIBERSORT algorithm in the training set. (B) The correlation heatmap between 7 diagnostic genes and 20 immune cells, with pink indicating positive correlation and purple indicating negative correlation.

**FIGURE 10:**
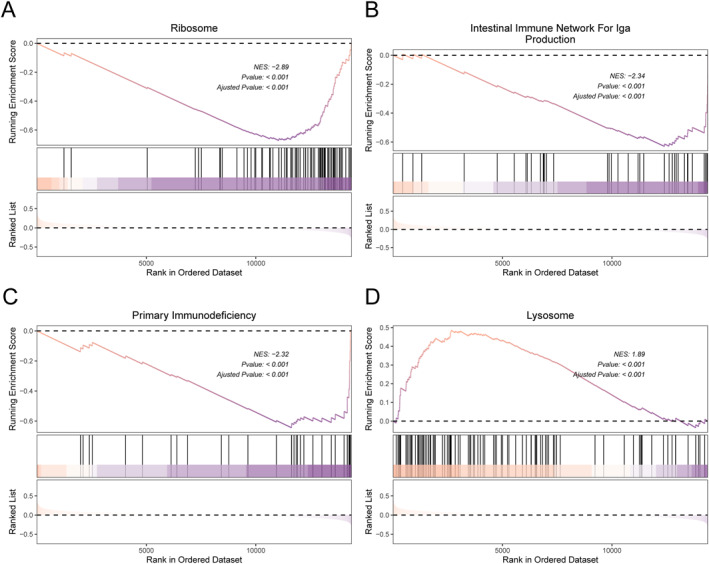
GSEA analysis between MDD and Normal group. The top four pathways with the most significant GSEA differences in genes between MDD and Normal samples.

**FIGURE 11:**
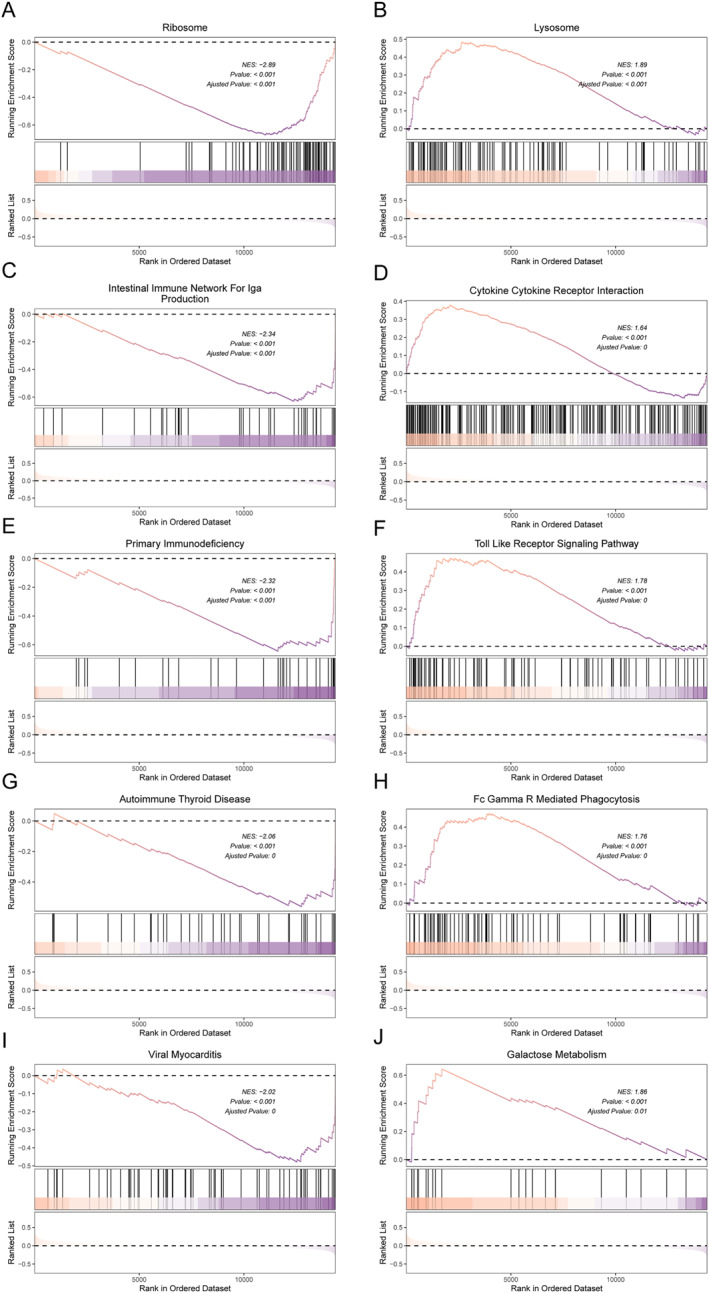
GSEA analysis of signal pathway between MDD and Normal group. GSEA inhibition (A, C, E, G, I) and activation (B, D, F, H, J) of the top 5 pathways of differentially expressed genes between MDD and Normal samples.

The CIBERSORT immune cell infiltration evaluation was conducted on the training set of MDD and normal samples. The results showed that there were significant differences in the content of multiple immune cells between MDD samples and normal samples, as shown in Figure 9A, and the four diagnostic genes were significantly correlated with the content of most immune cells in the 20 types of immune cells, as shown in Figure 9B. Meanwhile, the GSEA results between MDD and normal samples showed that the four pathways with the most significant differential enrichment between subtypes were the ribosome as shown in Figure 10A, the intracellular immune network for Iga production as shown in Figure 10B, primary immunodeficiency as shown in Figure 10C, and the lymphosome as shown in Figure 10D. The results of inhibiting and activating five pathways each were also presented, as was shown in Figure 11.

### 3.6 PPI Construction of Diagnostic Genes and Prediction of Small‐Molecule Drugs

**FIGURE 12:**
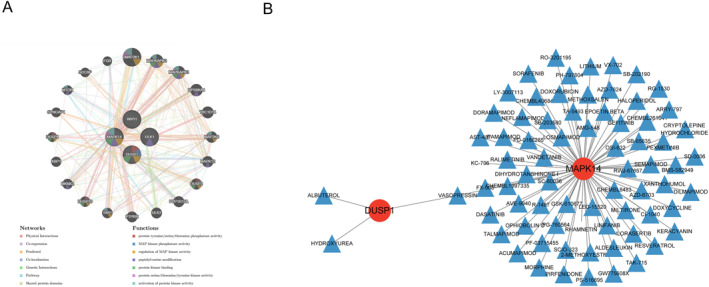
PPI construction of diagnostic genes and prediction of small‐molecule drugs. (A) Protein‐protein interaction network for diagnostic genes. (B) The interaction network between diagnostic genes and drugs.

First, a diagnostic gene network was constructed using the GeneMANIA database, as was shown in Figure 12A, with the inner circle representing diagnostic genes and the outer circle representing genes that interact with diagnostic genes. Different colours represent different functions. Then, a diagnostic iron death gene drug network was constructed using the DGIdb database, as was shown in Figure 12B, with red dots representing genes and blue triangles representing drugs. The prediction results showed that the small‐molecule drugs of ULK1 and MAPK14 amounts to 3 and 72, respectively.

## 4 Discussion

MDD is a complex psychiatric disorder with a lifetime prevalence of approximately 16% [15]. It is characterised by a range of symptoms including persistent sadness, loss of interest, and feelings of worthlessness [15]. The aetiology of MDD is multifactorial, involving genetic, environmental, and psychological factors. Depression was found to be substantially linked to worsening of the metabolic syndrome, increased incidence of diabetes, and increased incidence and severity of cardiovascular disease and events. It was also linked to an increased incidence of dementia and Alzheimer's disease as well as cognitive decline in people with pre‐existing diseases [16]. Newly discovered biomarkers associated with MD will provide insights into its molecular mechanisms. Ferroptosis is one of orientation potentially to be biomarkers facilitate MDD diagnosis and treatment.

This study analysed the differential expression of ferroptosis genes between MDD and normal samples. After batch effect processing and gene differential expression analysis, 1878 differentially expressed genes were identified, and several core modules were determined using WGCNA analysis. Further analysis revealed the widespread distribution of 17 differentially expressed ferroptosis‐related genes on chromosomes and significant expression differences in MDD. Finally, three algorithms obtained four diagnostic genes, ULK1, MAPK14, WIPI1, and DUSP1, using logistic regression to construct an MDD diagnostic model, it was found that the AUC values of the ROC curves for both the training and validation sets were greater than 0.7. Previous studies showed that these genes have been implicated in the pathophysiology of depression through their roles in cellular stress response, autophagy, and MAPK signalling, which are disrupted in MDD.

ULK1 (Unc‐51 Like Autophagy Activating Kinase 1) is a serine/threonine kinase that plays a critical role in the initiation of autophagy. ULK1 enhances mitophagy by stabilising the BNIP3 protein and phosphorylating S17, which stimulates interaction with LC3 [17]. Autophagy is a cellular process for the degradation and recycling of damaged proteins and organelles, which is dysregulated in various neuropsychiatric disorders, including depression. Ulk1‐mediated autophagy plays a beneficial effect in the reduction of neuropsychiatric manifestations following ethanol consumption [18]. Abnormal expression of ULK1 has been observed in depression, suggesting its potential role in the pathogenesis of the disorder [19]. Ferroptosis is an autophagy‐dependent cell death associated with metabolic failure and oxidative stress, demanding ongoing surveillance and future therapy development for oxidative stress‐related diseases such as neurological disorders [20]. Autophagy can mitigate iron metabolism dysregulation and lipid peroxidation, thereby modulating ferroptosis. For example, autophagy can reduce iron accumulation by clearing ferritin, or reduce lipid peroxidation by degrading lipids. ULK1 promotes mitochondrial autophagy by phosphorylating BNIP3, which is a regulatory protein of mitochondrial autophagy. Phosphorylated BNIP3 can bind to LC3 family proteins, promoting mitochondrial autophagic degradation. ULK1 affects ferroptosis by regulating the stability of BNIP3. ULK1 can increase the protein level of BNIP3 by inhibiting its proteasomal degradation, thereby promoting mitochondrial autophagy [17]. This effect is particularly evident under low oxygen conditions, as low oxygen can induce the expression of BNIP3 and promote the interaction between ULK1 and BNIP3. The effect of ULK1 inhibitors ULK‐101 can inhibit the kinase activity of ULK1, reduce the protein level of BNIP3, and thus inhibit mitochondrial autophagy. This suggests that ULK1 inhibitors may regulate ferroptosis by affecting mitochondrial autophagy. Overall, ULK1 affects the occurrence and development of ferroptosis by regulating autophagy processes, particularly mitochondrial autophagy. The activation of ULK1 can promote autophagy and mitochondrial clearance while the inhibition of ULK1 may lead to iron metabolism disorders and the accumulation of lipid peroxidation, thereby affecting ferroptosis. Furthermore, ULK1 has been identified as a potential therapeutic target for depression, with its modulation showing promise in preclinical models. Serine/threonine‐protein kinase ULK1 is one of the experimentally confirmed or predicted interactions with well‐known MDD‐related protein targets [21]. ULK1 was a promising therapeutic target within the autophagy framework, ULK1/Beclin‐1 initiation complex act as targeted autophagy modulation as a revolutionary pathway for developing more effective and personalised interventions for depressive disorders [22]. ULK1 was downregulated in frontal cortex (FC) and hippocampus (Hp) of male depression model rats exposed to chronic mild stress [23]. Ketamine (10 mg/kg) have played short‐versus long‐term antidepressant, cognitive, and psychotic effects on chronic unpredictable stress (CUS) rat model of depression by deactivated Unc‐51‐like autophagy activating kinase 1 (ULK1), and other autophagy markers [24]. Therefore, these experimental evidences demonstrated that ULK1 is a promising effective biomarker and target for depression treatment.

As a master regulator of autophagy initiation, inhibition of ULK1 may influence ferroptosis in MDD through several mechanisms. As previously noted, ULK1 facilitates mitophagy by stabilising and phosphorylating BNIP3 [17]. Within the context of MDD, dysregulated mitophagy could exacerbate neuronal damage by disrupting mitochondrial quality control and iron homoeostasis. Thus, pharmacological inhibition of ULK1 (using compounds such as ULK‐101 or SBI‐0206965) might restore mitophagic balance, limit iron accumulation from degraded mitochondria, and ultimately attenuate lipid peroxidation and ferroptotic cell death. These insights are consistent with our observation that ULK1 is upregulated in MDD, implying a pathogenic role that could be targeted therapeutically. Although ULK1 inhibitors have primarily been investigated in oncology, our findings highlight their potential relevance for MDD treatment. Repurposing such agents for neurological disorders represents a promising strategy, though challenges remain—particularly in achieving sufficient blood‐brain barrier penetration and maintaining selectivity to preserve basal autophagy in healthy neurons. Future research should prioritise evaluating these inhibitors in preclinical models of depression, examining their effects on ferroptosis markers (e.g., GPX4, PTGS2, lipid ROS), synaptic integrity, and depression‐related behaviours.

MAPK14, also known as p38, is a mitogen‐activated protein kinase that regulates various cellular processes including stress response, inflammation, and apoptosis [25]. It has been implicated in the pathophysiology of depression, with its activation being associated with neuronal damage and depressive symptoms [26]. The AKT and MAPK signalling pathways in the hippocampus indicate the pathophysiology of depression in four stress‐induced models [27]. Pharmacological inhibition of MAPK14 has been shown to ameliorate depressive‐like behaviours in animal models, highlighting its potential as a therapeutic target [25]. The activation of the SLC7A11/GPX4 ferroptotic pathway in the haemorrhagic shock rat model was shown by immunohistochemistry, which also revealed a substantial rise in the expression levels of CD44 and MAPK14.

The p38 MAPK pathway, encoded by MAPK14, plays a central role in cellular stress responses and neuroinflammation, each of which contributes to MDD pathogenesis. Activation of p38 may facilitate ferroptosis through phosphorylation of downstream effectors such as ATF4, which regulates genes involved in iron metabolism (e.g., TFRC) and lipid peroxidation. Additionally, p38 signalling stimulates the production of pro‐inflammatory cytokines (e.g., IL‐1β, TNF‐α), potentially creating a feed‐forward loop that amplifies both inflammation and ferroptosis. Notably, several p38 inhibitors (e.g., losmapimod, talmapimod, SB203580) have already entered clinical trials for inflammatory conditions such as rheumatoid arthritis and COPD [28, 29]. While their application in CNS disorders has been limited by poor bioavailability, our data provide a compelling rationale for testing these compounds in MDD subgroups with elevated inflammatory or ferroptosis biomarkers. Adopting a precision medicine framework, future clinical trials could enrich for patients with evidence of immune activation, offering a direct translational route for interventions targeting MAPK14.

WIPI1 (WD Repeat Domain, Phosphoinositide‐Interacting Protein 1) is involved in the regulation of autophagy and has been linked to depression. WIPI1 is critical for the formation of autophagosomes, and its dysregulation can lead to the accumulation of damaged cellular components, which may contribute to the development of depressive symptoms [30]. The role of WIPI1 in depression is an emerging area of research, with preliminary findings suggesting its potential involvement in the disorder's pathophysiology.

The upregulation of WIPI1 in MDD may indicate compensatory autophagy flux dysregulation or excessive autophagic activity contributing to neuronal impairment. Although direct WIPI1 inhibitors are unavailable, its function depends on PI3P generation by the Class III PI3K (PI3KC3/VPS34) complex. Inhibiting PI3KC3—using compounds such as wortmannin, LY294002, or VPS34‐IN1—represents an indirect therapeutic strategy to normalise WIPI1‐mediated autophagosome synthesis and mitigate ferroptosis, wherein aberrant autophagy promotes iron release and lipid peroxidation via degradation of ferritin and other protective components. However, clinical translation remains challenging due to poor specificity and pharmacokinetics of existing PI3KC3 inhibitors, though more selective, brain‐penetrant variants are under investigation, primarily in oncology. Precise targeting is essential to avoid disrupting constitutive autophagy; future efforts should develop allosteric or partial modulators of WIPI1 activity while near‐term approaches may focus on biomarker‐guided stratification of MDD patients for indirect pathway modulation.

DUSP1 (Dual Specificity Phosphatase 1) is a phosphatase that inactivates MAPKs, including ERK, JNK, and p38. It has been implicated in the regulation of the stress response and has been found to be differentially expressed in the hippocampus of rats subjected to the forced swim test, a widely used animal model of depression [31]. Overexpression of DUSP1 has been observed in postmortem brain tissues of MDD patients, suggesting its potential role in the pathophysiology of depression. DUSP1 protein levels were increased in the ventrolateral orbital cortex of chronic unpredictable mild stress rats [32].

Thus, we analysed the correlation between ULK1, MAPK14, WIPI1, and DUSP1 with infiltrating immune cells, and found that these four FRGs were significantly associated with immune cells, indicating an interaction between ferroptosis and immune response in MDD. Ferroptosis affects the immune system through various pathways such as activating immune responses, causing damage to the nervous system, disrupting the HPA axis, and affecting immune cells, and may play a key role in the occurrence and development of depression [33, 34], such as, ferroptosis is inhibited by sestrin2, which reduces neuroinflammation and depressive‐like behaviours in CUMS animals [35].

DUSP1/MKP‐1 functions as a key negative regulator of MAPK signalling, dephosphoryulating critical kinases such as p38 and JNK. Its upregulation in MDD may initially compensate for excessive neuroinflammatory signalling, yet sustained overexpression can disrupt MAPK homoeostasis and contribute to pathophysiology. Therapeutic strategies aimed at enhancing DUSP1 activity offer a promising approach by simultaneously suppressing multiple hyperactive MAPK pathways, thereby attenuating both neuroinflammation and MAPK‐driven ferroptotic cascades, including ATF4‐mediated iron metabolism dysregulation. Although no specific DUSP1 activators currently exist, existing drugs such as glucocorticoids and some antidepressants upregulate DUSP1, suggesting a shared mechanism underlying their efficacy. Translational efforts face challenges—particularly the non‐specific effects of glucocorticoids—but future development of selective DUSP1 enhancers or allosteric activators, combined with biomarker‐guided patient stratification, may enable precise targeting of inflammatory and stress pathways in MDD, advancing beyond conventional monoamine‐based therapeutics.

The observed correlation between FRGs and immune cell infiltration (e.g., monocytes, CD8+ T cells) suggests ferroptosis may modulate neuroinflammatory pathways in MDD. This aligns with prior evidence linking IL‐6 and TNF‐α to synaptic plasticity deficits [35, 36]. On the other hand, suppression results in a decrease in natural killer cell activity, which suggests ongoing immunological responses. Immune genes and depression are linked in part by genetic differences. Anti‐inflammatory therapy is one possible treatment approach that highlights the pathophysiology of depression and the importance of the immune system in future treatments [37, 38]. In this study, the present findings reveals that the expressions of ULK1, MAPK14, WIPI1, and DUSP1 were associated with the immune system, thereby participated in the pathogenesis of depression and could provide potential targets for future treatments.

This study analysed the differential expression of ferroptosis‐related genes ferroptosis‐related genes between depression (MDD) and normal samples. After batch effect processing and gene differential expression analysis, 1878 differentially expressed genes were identified, and several core modules were determined using WGCNA analysis. Further analysis revealed the widespread distribution of 17 differentially expressed ferroptosis‐related genes on chromosomes and significant expression differences in MDD. These genes are enriched in macroscopic autophagy and nutrient response pathways. Four diagnostic genes (ULK1, MAPK14, WIPI1, and DUSP1), and a MDD diagnostic model was constructed, which demonstrated good predictive performance in both the training and validation sets. Microenvironment assessment showed significant differences in immune cell content between MDD samples and normal samples, and diagnostic genes were associated with immune cell content. GSEA analysis revealed significant differences in signalling pathways between MDD and normal samples. Furthermore, we constructed a diagnostic gene interaction network and identified potential small‐molecule therapeutics, providing new insights into the molecular mechanisms and treatments of MDD. In conclusion, ULK1, MAPK14, WIPI1, and DUSP1 are hub genes with established roles in cellular stress response and autophagy. Their dysregulation has been implicated in the pathophysiology of MDD, providing novel insights into the molecular mechanisms underlying the disorder.

In conclusion, we successfully constructed the novel diagnostic model for MDD based on a distinct set of ferroptosis‐related hub genes (ULK1, MAPK14, WIPI1, DUSP1), which overcomes the limitations of traditional biomarkers and provides a novel tool for the diagnosis of MDD. By employing WGCNA combined with machine learning algorithms such as LASSO, SVM‐RFE, and RF, we were able to screen key genes more effectively, thereby enhancing the reliability of the model. The study clarified that FRGs participate in the pathological process of MDD by regulating autophagy and immune infiltration, offering a new perspective for immunometabolic research. In addition, we predicted small molecule drugs, such as ULK1 inhibitors, which could potentially promote the application of translational medicine. These innovative points not only advance the understanding of the role of FRGs in MDD but also provide new directions for future research and treatment strategies. While our bioinformatic analyses revealed significant correlations between ferroptosis‐related genes and immune infiltration in MDD, these findings necessitate experimental validation.

## Author Contributions

Conception and design: Shenghui Huang and Hailong Li. Administrative support: Shenghui Huang. Provision of study materials or patients: Fei Feng, Shoupin xie, Yanyan Wan. Collection and assembly of data: Ping Tang, Yanping Ma, Xinhong Chen. Data analysis and interpretation: Yafeng Wang, Fan Zhang. Manuscript writing: Hailong Li. Final approval of manuscript: All authors.

## Funding

This study was supported by Gansu Province Traditional Chinese Medicine Research Project (GZKP‐2023‐2), Shenzhen Key Medical Discipline Construction Fund (No. SZXK062), and the Joint Research Fund of Provincial Science and Technology Programme of Gansu Province (23JRRA1536).

## Ethics Statement

The authors have nothing to report.

## Consent

All authors agreed to publication of this paper.

## Conflicts of Interest

The authors declare no conflicts of interest.

## Data Availability

All data are available when requested by readers.
